# Multiresistant ST59-SCC*mec* IV-t437 clone with strong biofilm-forming capacity was identified predominantly in MRSA isolated from Chinese children

**DOI:** 10.1186/s12879-017-2833-7

**Published:** 2017-11-25

**Authors:** Xin Yang, Suyun Qian, Kaihu Yao, Lijuan Wang, Yingchao Liu, Fang Dong, Wenqi Song, Jinghui Zhen, Wei Zhou, Hong Xu, Hongyan Zheng, Wenting Li

**Affiliations:** 1 Pediatric Intensive Care Unit, Beijing Children’s Hospital, Capital Medical University, National Center for Children’s Health, No.56 Nan-Li-Shi Road, Beijing, 100045 China; 2 MOE Key Laboratory of Major Diseases in Children, National Key Discipline of Pediatrics (Capital Medical University), National Clinical Research Center for Respiratory Diseases, Beijing Key Laboratory of Pediatric Respiratory Infection Diseases, Beijing Pediatric Research Institute, Beijing Children’s Hospital, Capital Medical University, National Center for Children’s Health, Beijing, 100045 China; 3 Bacteriology Laboratory, Beijing Children’s Hospital, Capital Medical University, National Center for Children’s Health, Beijing, 100045 China

**Keywords:** *Staphylococcus aureus*, Biofilm, Antimicrobial resistance, Clonal lineage, Pediatrician, China

## Abstract

**Background:**

This study aimed to investigate the clinical and molecular epidemiology and biofilm formation of *Staphylococcus aureus* (SA) isolated from pediatricians in China.

**Methods:**

SA strains were isolated from Beijing Children’s hospital from February 2016 to January 2017. Isolates were typed by multilocus sequence typing (MLST), *spa* and SCC*mec* typing (for Methicillin-resistant SA [MRSA] only). Antimicrobial susceptibility testing was performed by agar dilution method except sulphamethoxazole/trimethoprim (E-test method). Biofilm formation and biofilm associated genes were detected.

**Results:**

Totally 104 children (41 females and 63 males; median age, 5.2 months) were enrolled in this study, in which 60 patients suffered from MRSA infection. Among the 104 cases, 54.8% were categorized as community associated SA (CA-SA) infections. The children under 3 years were more likely to occur CA-SA infections compared with older ones (*P* = 0.0131). ST59-SCC*mec* IV-t437 (61.7%) was the most prevalent genotype of MRSA, and ST22-t309 (18.2%), ST5-t002 (9.1%), ST6-t701 (9.1%), ST188-t189 (9.1%) were the top four genotypes of methicillin-sensitive SA (MSSA). All the present isolates were susceptible to linezolid, vancomycin, trimethoprim-sulfamethoxazole, mupirocin, tigecyclin, fusidic acid. No erythromycin-susceptible isolate was determined, and only a few isolates (3.8%) were identified as susceptible to penicillin. Multi-drug resistant isolates were reponsible for 83.8% of the ST59-SCC*mec* IV-t437 isolates. The isolates with strong biofilm formation were found in 85% of MRSA and 53.2% of MSSA, and in 88.7% of ST59-SCC*mec* IV-t437 isolates. Biofilm formation ability varied not only between MRSA and MSSA (*P =* 0.0053), but also greatly among different genotypes (*P* < 0.0001). The prevalence of the biofilm associated genes among ST59-SCC*mec* IV-t437 clone was: *icaA* (100.0%), *icaD* (97.3%), *fnbpA* (100.0%), *fnbpB* (0), *clfA* (100%), *clfB* (100%), *cna* (2.7%), *bbp* (0), *ebpS* (88.5%), *sdrC* (78.4%), *sdrD* (5.4%), and *sdrE* (94.5%)*.*

**Conclusions:**

These results indicated strong homology of the MRSA stains isolated from Chinese children, which was caused by spread of multiresistant ST59-SCC*mec* IV-t437 clone with strong biofilm formation ability. The MSSA strains, in contrast, were very heterogeneity, half of which could produce biofilm strongly.

**Electronic supplementary material:**

The online version of this article (10.1186/s12879-017-2833-7) contains supplementary material, which is available to authorized users.

## Background

*Staphylococcus aureus* (SA) is an important Gram-positive pathogen which can cause diseases ranging from minor to potentially life-threatening community associated and hospital-associated infections, such as skin and soft tissue infections (SSTIs), bacteremia, pneumonia, osteomyelitis and endocarditis [1]. SA also has the ability to form biofilm in biological and indwelling medical devices surfaces [2]. The successful eradication of SA infection in patients become difficult once biofilm formed, since biofilm can protect SA from the damage of antibiotics, host immune system, and so on [2]. In addition, Savage et al. found that SA biofilms could promote horizontal spread of antibiotic resistance determinants, which were mainly through increasing the frequency of plasmid transfer events by both conjugation and mobilization [3]. Thus, biofilm forming ability of SA has drawn considerable interest from researchers over the past decades.

Biofilm formation can be divided into at least three major stages: initial attachment, biofilm maturation, and dispersal [4]. Initial attachment is a crucial stage of transition from an individual planktonic cell to a biofilm. Attachment is mediated mainly through a family of surface proteins, referred to as microbial surface components recognizing adhesive matrix molecules (MSCRAMMs), such as clumping factor A (ClfA), clumping factor B (ClfB), elastin binding protein (EbpS), serine-aspartate repeat protein C (SdrC), SdrD, SdrE, bone sialoprotein-binding protein (Bbp, isoform of SdrE), fibronectin-binding proteins A (FnBPA) and B (FnBPB), collagen adhesin (Cna) [5]. During the stages of biofilm maturation, multilayered biofilm formation is related to the production of polysaccharide intercellular adhesin (PIA), which is synthesized by the enzymes encoded by the intercellular adhesion (*ica*) operon, mainly including *icaR* (intercellular adhesion regulator) and *icaA*, *B*, *C*, and *D* [6]. Among these genes, *icaA* and *icaD* are most extensively studied and play a more important role in the biofilm formation than other genes [7].

Although many studies have reported the phenotypic and genotypic basis for biofilm production in SA clinical strains isolated from different infectious diseases and different countries [8–10], little is known regarding the biofilm formation ability of SA clinical strains isolated from Chinese, especially children. According to our knowledge, only the prevalence of adhesion genes was ever reported among SA strains isolated from children in china, but these studies didn’t assess the biofilm formation ability of bacteria [11, 12].

Considering the adverse effect of biofilm formation on SA mediated infectious diseases [2, 3] as well as shifts of major clones in a given region over time [13], the present study aimed to investigate the genotype characteristics, antimicrobial susceptibility, biofilm-forming ability and the prevalence of biofilm associated genes among SA clinical strains, which were collected from the biggest tertiary-care teaching hospital for children in Beijing, China.

## Methods

### Bacteria isolates

This study was performed in Beijing Children’s Hospital in China. It was reviewed and approved by the Ethics Committee of Beijing Children’s Hospital affiliated to Capital Medical University. No ethical problems existed in this study.

Once SA was detected from Bacteriology Laboratory in our hospital, the isolates were collected and stored, but bacteria isolated from throat swab, vaginal secretions, and defecate were not included. Only one strain was included from each patient. A total of 209 isolates were collected during the studied period. Of the 209 isolates, 19 were collected from outpatients (lack of epidemiological information), 86 were identified as colonizing strains, and only 104 were considered to have caused clinical infections. Thus, the 104 pathogenic bacteria were selected for further study. These strains were isolated from several clinical sources, including respiratory tract (27 form sputum, 15 from bronchial alveolar lavage fluid), skin and soft tissue (11 from pus, 8 from secretions, 13 from secretions of omphalitis, 5 from eye secretions), sterile sites (20 from blood, 2 from joint effusion and 2 from pleural effusion), and pipe end (1 isolate). SA infections were categorized as hospital associated (HA) or community associated (CA) according to the definitions established previously [14].

The identification of the SA isolates was performed by colony morphological characteristics, coagulase test, and nuc gene detection. The MRSA isolates were screened with cefoxitin discs and were confirmed by detecting the carrying situation of the *mecA* gene by polymerase chain reaction (PCR) [11]. All strains were stored at −80 °C until use.

### Extraction of genomic DNA

A typical colony was cultivated on blood agar at 37 °C for 24 h. Bacteria genomic DNA was extracted using Nucleic Acids Isolation & Purification kit (Saibaisheng gene technology Ltd., China) according to the manufacturer’s instructions.

### Molecular genotyping analysis

MLST was performed as described by Enright et al. previously [15]. The seven housekeeping gene (*arcC, aroE, glpF, gmk, pta, tpi and yqiL*) sequences were compared with known alleles from the MLST database (http://saureus.mlst.net/), and the allelic profiles (allele numbers) and ST types were determined based on the database.

The the polymorphic X region of *spa* gene was amplified as previously described [16], and the *spa* type was determined by submitting the sequencing data to the SA *spa* type database (http://spaserver.ridom.de).

For Methicillin-resistant SA (MRSA) isolates, the SCC*mec* types were determined using a multiplex PCR as previously described [17]. The following MRSA strains were used as a positive control for SCC*mec* types: NCTC10442 (SCC*mec* I), N315 (SCC*mec* II), 85/2082 (SCC*mec* III), JCSC4744 (SCC*mec* IV), IMVS 67(SCC*mec* V).

### Antimicrobial susceptibility testing

The susceptibility of the isolates against penicilin G, cefuroxime, gentamicin, rifampin, ciprofloxacin, clindamycin, erythromycin, chloramphenicol, tetracycline, linezolid, vancomycin, mupirocin, tigecycline and fusidic acid were tested with the agar dilution methods. Susceptibility to sulphamethoxazole/trimethoprim was determined by E-Test method. Minimum inhibitory concentration for tigecycline and fusidic acid were interpreted using European Committee on Antimicrobial Susceptibility Testing (EUCAST) breakpoints for *Staphylococcus* spp. [18]. The MIC of other antibiotics were interpreted using the Clinical and Laboratory Standards Institute (CLSI) breakpoints for *Staphylococcus* spp. [19]. ATCC29213 was used as the quality control. For MRSA, multi-drug resistance (MDR) was defined as isolates resistant to ≥ 3 classes of non-β-lactam antimicrobials [20], whereas MDR was defined as isolates resistant to ≥ 3 classes of antibiotics including β-lactam antibiotics for Methicillin-sensitive SA (MSSA).

### Detection of biofilm associated genes

The following genes were detected using PCR assays: *icaA*, *icaD*, *fnbpA*, *fnbpB*, *clfA*, *clfB*, *cna*, *bbp*, *ebpS*, *sdrC*, *sdrD*, *sdrE*. The primers and amplification conditions of these genes were previously described by Darwish et al. (*icaA*, *icaD*) [21], Otsuka et al. (*fnbpA*) [22], Tristan et al. (*fnbpB*, *clfA*, *clfB*, *cna*, *bbp*) [23], Campbell et al. (*ebpS*, *sdrC*, *sdrD*) [24], and Peacock et al. (*sdrE*) [25]. N315 was used as positive control for *icaA*, *icaD, ebpS, sdrC,sdrD, and sdrE*; RN4220 was used as positive control for *fnbpA*, *fnbpB*, *clfA*, *clfB*; ATCC25923 was used as positive control for *cna* and *bbp* [26, 27]. The presence and size of the PCR products were confirmed by electrophoresis on 1.5% agarose gels.

### Biofilm formation assays

Biofilm forming ability was assessed using tissue culture plate method (TCP), as described by Xu et al. [28], with slight modification. Briefly, All MRSA strains were grown in tryptic soy broth (TSB) (OXOID, USA) containing 0.25% glucose overnight at 37 °C. Bacterial concentrations were adjusted to a concentration of 0.5 on the McFarland scale (~1.5 × 10^8^ CFU/mL), and diluted in TSB containing 0.25% glucose to a final concentration of 10^6^ CFU/ml. The biofilm assay was performed in 96-well flat-bottom plates (Corning, USA) at 37 °C for 48 h. Because 48 h of growth has been optimal for SA, and biofilms are sufficiently mature at this time point [29, 30]. Subsequently, wells were washed twice 0.9% sodium chloride, and fixed by methanol for 15 min. After air dried, wells were stained with 0.1% crystal violet for 5 min. The microtiter plate was then rinsed with PBS and air dried, and the stained biofilm was resuspended for quantification in 33% glacial acetic acid. The optical density (OD) of each stained well was measured at 590 nm using an CLARIOstar Microplate reader (BMG LABTECH, Germany). Each isolate was tested in three repetition. The negative control wells contained broth only.

The cut-off OD value (ODc) was defined as the arithmetic mean of the absorbance of negative controls with three times addition of standard deviation. The following classification was applied for the determination of biofilm formation: no biofilm production (OD ≤ ODc), weak biofilm production (WBF, ODc < OD ≤ 2ODc, WBF), moderate biofilm production (2ODc < OD ≤ ODc, MBF), and strong biofilm production (4ODc < OD, SBF).

### Statistical analysis

SAS JMP Statistical Discovery v11.0 was used for statistical analysis. Categorical variables were analyzed using Chi-squared (χ2) test or Fisher’s exact test. The OD values used to assess the biofilm formation didn’t coincided with normal distribution in any cases, so Wilcoxon rank sum test was used to compare the biofilm formation ability between two groups. In addition, when compared the biofilm formation ability among three or more groups, Kruskal–Wallis test followed by Steel–Dwass test were used. *P* < 0.05 was considered statistically significant.

## Results

### Clinical characteristics

**Fig. 1:**
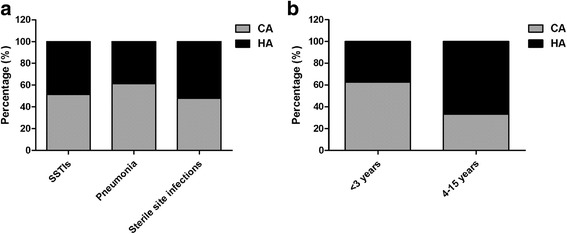
The modes of acquisition among different infections (**a**) and different age groups (**b**)

A total of 104 children (41 females and 63 males; median age, 5.2 months) were enrolled in this study, and 60 patients suffered from MRSA infection. Their clinical characteristics were shown in Table 1. Approximately 74.0% (74/104) of the patients were less than 3 years old. By CDC criteria, 54.8% (57/104) were categorized as community associated infections, and 45.2% (47/104) were categorized as hospital associated infections. The modes of acquisition (hospital vs. community) were similar among MRSA and MSSA (Table 1) and different sites of infections (Fig. 1a). Children under 3 years were more likely to occur community associated infections compared with older children (*P* = 0.0131) (Fig. 1b). SSTIs (35.58%, 37/104) and pneumonia (42.3%, 44/104) were the top two sites of SA infections. Sterile site infections also accounted for 22.1% (23/104) of total SA infections. The incidence of MRSA was significantly different among different infectious diseases (*P* = 0.0031). Patients with SSTIs were more likely to suffer MRSA infections (75.68%, 28/37), and patients with bloodstream infections were more likely to suffer MSSA infections (71.43%, 15/21). Thirty-four patients co-infected with other organisms. No significant differences were found between MRSA and MSSA in terms of laboratory examination and hospitalization conditions (*P* > 0.05).

**Table 1:** Pathogen and patient characteristics

Characteristics	Total	MRSA	MSSA	*P* value
Patients	104	60	44	
Patient characteristics				
Female sex, n (%)	41 (39.4)	28 (46.7)	13 (29.5)	0.1044
Age (months), median (IQR^a^)	5.2 (49.6)	3.9 (57.5)	8.3 (49.4)	0.424
Age distribution				0.7883
≤ 28 day	31 (29.8)	20 (33.3)	11 (25.0)	
29 day-3 years	46 (44.2)	25 (41.7)	21 (47.7)	
4–6 years	10 (9.6)	5 (8.3)	5 (11.4)	
7–15 years	17 (16.4)	10 (16.7)	7 (15.9)	
Origin				1.000
Community associated	57 (54.8)	33 (55.0)	24 (54.5)	
Hospital associated	47 (45.2)	27 (45.0)	20 (45.5)	
Disease				0.0031
Skin and soft tissue infection	37 (35.58)	28 (46.7)	9 (20.5)	
Pneumonia	44 (42.3)	24 (40.0)	20 (45.5)	
Bloodstream infection	21 (20.2)	6 (10.0)	15 (34.1)	
Bone and joint infection	2 (1.9)	2 (3.3)	0	
Laboratory examination				
White cell count-Median (IQR) (10^9^/L)	13.7 (9.88)	13.9 (8.7)	13.3 (12.4)	0.5834
Neutrophil count-Median (IQR) (10^9^/L)	7.9 (10.74)	8.7 (10.4)	7.2 (11.9)	0.7365
Neutrophils percentage-Median (IQR)	61.6 (33.3)	62.1 (30.0)	60.9 (36.8)	0.7107
C-reactive protein-Median (IQR) (mg/L)	18.5 (51.5)	14.0 (66.0)	24.0 (50.0)	0.8506
Co-infection^b^	34.0 (32.7)	18.0 (30.0)	16 (36.4)	0.5308
Hospitalization				
Hospital days-median (IQR)	13 (11.0)	12 (10)	13 (10.8)	0.9056
Intensive care unit (ICU) admission	26 (25.0)	12 (20.0)	14 (31.8)	0.1789
MLST				<0.0001
5	5 (4.8)	0	5 (11.4)	
6	5 (4.8)	1 (1.7)	4 (9.1)	
7	4 (3.8)	0	4 (9.1)	
22	11 (10.6)	2 (3.3)	9 (20.5)	
25	4 (3.8)	0	5 (11.4)	
59	49 (47.1)	46 (76.7)	3 (6.8)	
188	4 (3.8)	0	4 (9.1)	
398	5 (4.8)	0	5 (11.4)	
Others^c^	17 (16.3)	11 (18.3)	6 (13.6)	
*spa* type				<0.0001
t002	4 (3.8)	0	4 (9.1)	
t189	4 (3.8)	0	4 (9.1)	
t309	12 (11.5)	3 (5.0)	9 (20.5)	
t437	41 (39.4)	39 (65.0)	2 (4.5)	
t441	4 (3.8)	4 (6.7)	0	
t701	4 (3.8)	0	4 (9.1)	
Others^d^	35 (33.7)	14 (23.3)	30 (68.2)	
SCC*mec* type (only for MRSA)				/
IV	51 (85.0)	51 (85.0)	/	
V	6 (10.0)	6 (10.0)	/	
NT^e^	3 (5.0)	3 (5.0)	/	

^a^IQR, interquartile range

^b^Including bacteria (*Pertussis*, *Acinetobacter baumanii*, *Klebsiella pneumoniae*, *Pseudomonas aeruginosa*, *Escherichia coli*, *Mycobacterium Tuberculosis*, *Streptococcus pneumoniae*, *Streptococcus pyogenes*, *Haemophilus influenzae*, *Enterobacter cloacae*), fungus (*Candida albicans*, *Candida krusei*), virus (*Respiratory syncytial virus*, *Influenza A virus*, *Adenovirus*, *Cytomegalovirus*) and *Mycoplasma pneumoniae*

^c^The other MLSTs were ST1 (1 isolate), ST30 (1 isolate), ST97 (1 isolate), ST121 (1 isolate), ST338 (2 isolates), ST896 (1 isolate), ST1224 (1 isolate), ST1821 (1 isolate) in the MRSA group, and ST1 (1 isolate), ST15 (3 isolates), ST121 (1 isolate), ST950 (1 isolate) in the MSSA group

^d^The other spa types were t021 (1 isolate), t114 (3 isolates), t163 (1 isolate), t172 (2 isolates), t267 (1 isolate), t1751 (1 isolate), t3515 (1 isolate), t3590 (1 isolate), t4549 (1 isolate), t8860 (1 isolate), t12946 (1 isolate) in the MRSA group, and t034 (2 isolates), t078 (2 isolates), t084 (3 isolates), t091 (2 isolates), t127 (1 isolate), t163 (1 isolate), t167 (1 isolate), t310 (1 isolate), t571 (3 isolates), t660 (1 isolate), t796 (1 isolate), t1062 (1 isolate), t1818 (1 isolate), t2092 (1 isolate) in the MSSA group

^e^Nontypable

### Genotypic characterization

The genotypic characteristics of the bacteria were also shown in Table 1. A total of eighteen STs were identified. MRSA isolates showed 11 STs, and ST59 (76.7%, 46/60) was the most prevalent. The frequencies of the remaining STs were very low, ranging from 1% to 4%. MSSA strains showed 12 STs. The top three STs in MSSA were ST22 (20.5%, 9/44), ST5 (11.4%, 5/44) and ST398 (11.4%, 5/44). The frequencies of the remaining STs were ranging from 2.3% (1/44) to 9.1% (4/44).

The *spa* typing discriminated the 104 isolates into 31 *spa* types. The 60 MRSA isolates belonged to 14 *sp*a types. Among them, t437 (65%, 39/60) was the most prevalent, followed by t441 (4/60). The prevalence rates of the remaining *spa* types were ranging from 1.7% (1/60) to 5.0% (3/60). 20 spa types were found in 44 MSSA isolates. The most common *spa* types were t309 (20.5%, 9/44), t002 (9.1%, 4/44), t189 (9.1%, 4/44). The remaining *spa* types accounted for 2.2% (1/44) to 6.8% (3/44) of all MSSA isolates.

SCC*mec* typing for MRSA isolates showed that nearly 85% (51/60) isolates harbored SCC*mec* type IV, followed by SCC*mec* V (10%, 6/60). No isolate harbored SCC*mec* I, II or III. Besides, the SCC*mec* type of three isolates couldn’t be determined.

Combined analysis of MLST, *spa* and SCC*mec* types (for MRSA only) indicated that ST59-SCC*mec* IV-t437 (61.7%, 37/60) was the most prevalent clone among MRSA isolates. The top 4 genotypes of MSSA were ST22-t309 (18.2%, 8/44), ST5-t002 (9.1%, 4/44), ST6-t701 (9.1%, 4/44), ST188-t189 (9.1%, 4/44).

### Distribution of STs in different epidemiologic category and infections

**Fig. 2:**
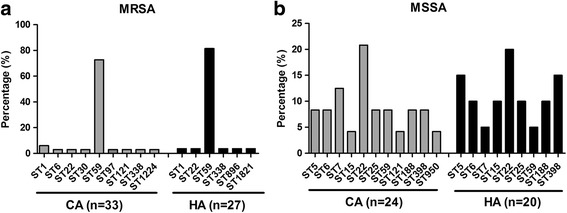
Prevalence of *Staphylococcus aureus* STs among different modes of acquisition. **a** Distribution of the STs among CA-MRSA and HA-MRSA isolates; **b** Distribution of the STs among CA-MSSA and HA-MSSA isolates

ST59 was the most prevalent clone both in CA-MRSA and HA-MRSA isolates, with a distribution of 72.7% (24/33) and 81.5%(22/27), respectively (Fig. 2a). No predominant STs were found in CA- and HA- MSSA isolates. ST22 was identified in 5 (20.8%) CA-MSSA and 4 (20%) HA-MSSA isolates (Fig. 2b).

The predominant clone in SSTIs and pneumonia was identified as ST59, which accounted for 62.2% (23/37) and 45.5% (20/44), respectively. Among sterile site infections, ST22 (30.4%, 7/23) was the most prevalent, followed by ST59 (26.1%, 6/23) (Additional file 1).

### Antimicrobial resistance

Antimicrobial susceptibility test results were shown in Table 2. All isolates in this study were susceptible to trimethoprim-sulfamethoxazole, linezolid, vancomycin, mupirocin, tigecyclin, fusidic acid. Only 2 isolates were resistance to rifampin. All isolates were non-susceptible to erythromycin, and nearly all isolates (96.2%, 100/104) were non-susceptible to penicillin. The non-susceptibility rates to cefuroxime, clindamycin, and tetracycline were significantly higher in MRSA than MSSA (*P* < 0.05). However, the non-susceptibility rate to gentamicin was significantly lower in MRSA than MSSA (*P* = 0.0069). About 76.7% (46/60) of MRSA and 77.3% (34/44) of MSSA were MDR isolates.

**Table 2:** Non-susceptibility rates of different genotypes of SA in pediatric population in China

	Isolates (n)	PEN	CXM	ERY	CLI	TET	GEN	CHL	RIF	CIP	SXT	LZD	VAN	MUP	TGC	FUS	MDR^a^
MRSA	60	96.7	38.4	100.0	83.3	40.0	5.0	63.3	3.3	20.0	0	0	0	0	0	1.7	76.7
ST59-SCC*mec* IV-t437	37	97.3	45.9	100.0	91.9	54.1	2.7	70.3	2.7	18.9	0	0	0	0	0	0	83.8
Others	23	95.7	26.1	100.0	69.6	82.6	8.7	52.2	4.3	21.7	0	0	0	0	0	4.3	65.2
MSSA	44	95.5	0	100.0	31.8	18.2	25.0	61.4	0	13.6	0	0	0	0	0	0	77.3
ST5-t002	4	75.0	0	100.0	75.0	25.0	75.0	50.0	0	0	0	0	0	0	0	0	75.0
ST6-t701	4	100.0	0	100.0	0	0	0	75.0	0	0	0	0	0	0	0	0	75.0
ST22-t309	8	100.0	0	100.0	12.5	12.5	0	37.5	0	0	0	0	0	0	0	0	62.5
ST188-t189	4	100.0	0	100.0	25.0	25	25.0	75.0	0	25.0	0	0	0	0	0	0	75.0
Others	24	95.8	0	100.0	37.5	20.8	29.2	66.7	0	20.8	0	0	0	0	0	0	83.3
Total	104	96.2	22.1	100.0	61.5	30.8	13.4	62.5	1.9	17.3	0	0	0	0	0	0	85.6
*P* value		1.000	<0.0001	–	<0.0001	0.0193	0.0069	0.8408	0.5071	0.4431	–	–	–	–	–	1.000	1.000

*PEN* Penicillin, *CXM* Cefuroxime, *ERY* Erythromycin, *CLI* Clindamycin, *TET* Tetracycline, *GEN* Gentamicin, *CHL* Chloramphenicol, *CIP* Ciprofloxacin, *RIF* Rifampin, *SXT* Trimethoprim-sulfamethoxazole, *LNZ* Linezolid, *VAN* Vancomycin, *MUP* Mupirocin, *TGC* Tigecycline, *FA* Fusidic acid

^a^ *MDR* multi-drug resistance. MDR-MRSA, resistant to ≥ 3 classes of non-β-lactam antimicrobials; MDR-MSSA, resistant to ≥ 3 classes of antibiotics including β-lactam antibiotics

The non-susceptibility rates of ST59-SCC*mec* IV-t437 to penicillin, cefuroxime, erythromycin, clindamycin, tetracycline, gentamicin, chloramphenicol, ciprofloxacin, rifampin were 97.3% (36/37), 45.9% (17/37), 100.0% (37/37), 91.9% (34/37), 54.1% (20/37), 2.7% (1/37), 70.3% (26/37), 18.9% (7/37) and 2.7% (1/37), respectively, and the MDR rate was 83.8% (31/37). The top three resistance phenotypes of this clone to non-β-lactam antimicrobials were ERY-CLI-TET-CHL (32.4%, 12/37), ERY-CLI-CHL (21.6%, 8/37), and ERY-CLI (10.8%, 4/37).

### Biofilm production

Table 3 showed the biofilm formation ability of MRSA and MSSA. Among 60 MRSA strains, 50 isolates (83.3%) showed SBF, 9 isolates (15.0%) showed MBF, 1 (1.67%) isolates showed WBF. Nearly 87.0% (40/46) of the ST59 strains, 86.3% (44/51) of SCC*mec* IV strains and 84.6% (33/39) of *spa* t437 type strains could form strong biofilm. Combined analysis of different genotypes showed that 83.8% (31/37) of strains belonging to ST59-SCC*mec* IV-t437 clone were strong biofilm former. All MSSA isolates tested were also attached at different levels (Table 2), 54.5% (24/44) exhibited SBF, 40.9% (18/44) exhibited MBF, and 4.5% (2/44) exhibited WBF. In addition, all ST188-t189 MSSA isolates showed SBF. The raw OD value of all isolates were shown in Additional file 2.

**Table 3:** Biofilm formation ability of MRSA and MSSA regarding to different genotypes

Genotype	Isolates (n)	WBF (n, %)	MBF (n, %)	SBF (n, %)	OD value (Median)
MRSA	60	1 (1.67)	9 (15.0)	50 (83.3)	0.68
MLST type					
ST59	46	0	6 (13.0)	40 (87.0)	0.72
Others	14	1 (7.1)	3 (21.4)	10 (71.4)	0.59
SCC*mec* type					
IV	51	0	7 (13.7)	44 (86.3)	0.69
V	6	0	1 (16.7)	5 (83.3)	0.59
NT*	3	1 (33.3)	1 (33.3)	1 (33.3)	0.22
*spa* type					
t437	39	0	6 (15.4)	33 (84.6)	0.67
Others	21	1 (4.8)	3 (14.3)	17 (80.9)	0.70
Combined genotype					
ST59-SCC*mec*IV-t437	37	0	6 (16.2)	31 (83.8)	0.67
Others	23	1 (4.4)	3 (13.0)	19 (82.6)	0.70
MSSA	44	2 (4.5)	18 (40.9)	24 (54.5)	0.42
MLST type					
5	5	0	1 (20.0)	4 (80.0)	0.65
6	4	0	2 (50.0)	2 (50.0)	0.36
7	4	0	1 (25.0)	3 (75.0)	0.84
22	9	0	4 (44.4)	5 (55.6)	0.42
25	4	1 (25.0)	2 (50.0)	1 (25.0)	0.26
188	4	0	0	4 (100.0)	1.14
398	5	0	5 (100.0)	0	0.34
Others	9	1 (11.1)	3 (33.3)	5 (55.6)	0.49
*spa* type					
t002	4	0	1 (25.0)	3 (75.0)	0.60
t189	4	0	0	4 (100.0)	1.1
t309	9	0	5	4	0.36
t701	4	0	2 (50.0)	2 (50.0)	0.36
Others	23	2	10	11	0.40
Combined genotype					
ST5-t002	4	0	1 (25.0)	3 (75.0)	0.60
ST6-t701	4	0	2 (50.0)	2 (50.0)	0.36
ST22-t309	8	0	4 (50.0)	4 (50.0)	0.37
ST188-t189	4	0	0	4 (100.0)	1.1
Others	24	2 (8.7)	11 (45.8)	11 (45.8)	0.38

^*^Nontypable

**Fig. 3:**
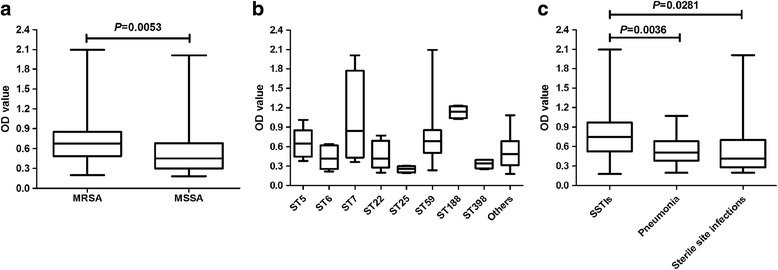
Biofilm formation assays of *Staphylococcus aureus*. **a** Comparison of the biofilm formation ability between MRSA and MSSA isolates. **b** Comparison of the biofilm formation ability of common genotypes. **c** Biofilm formation ability of *Staphylococcus aureus* isolated from pediatricians with different infections

Moreover, significant difference between MRSA and MSSA regarding biofilm formation ability was found (*P* = 0.0053) (Fig. 3a). We further compared the biofilm formation ability of different genotypes, and significant differences were found among them (*P* < 0.0001) (Fig. 3b), but no significant differences were found between any two groups (*P* > 0.05).

We further compared the biofilm formation ability of SA isolated from patients with different infections (Fig. 3c). Strains isolated from patients with SSTIs could product much higher biofilm than strains isolated from patients with pneumonia (*P* = 0.0036) and sterile site infections (*P* = 0.0281).

### Distribution of biofilm associated genes

Table 4 showed the prevalence of biofilm associated genes among MRSA and MSSA isolates. For MRSA, all isolates were positive for *icaA*, *fnbpA*, *clfA*, *clfB* and only one strain was *icaD* negative. The prevalence rates for *fnbpB*, *cna* and *bbp* were very low, their carrying rates were 3.3%, 10.0% and 1.7%, respectively. The prevalence rates for *ebpS*, *sdrC*, *sdrD*, *sdrE* were ranged from 20.0% to 91.7%, respectively.

**Table 4:** The prevalence of biofilm associated genes among MRSA and MSSA clinical isolates

Comined genotype	Isolates(n)	*icaA*	*icaD*	*fnbpA*	*fnbpB*	*clfA*	*clfB*	*cna*	*bbp*	*ebpS*	*sdrC*	*sdrD*	*sdrE*
MRSA	60	100.0	98.3	100.0	3.3	100.0	100.0	10.0	1.7	85.0	76.7	20.0	91.7
ST59-SCC*mec*IV-t437	37	100.0	97.3	100.0	0	100.0	100.0	2.7	0	88.5	78.4	5.4	94.5
Others	23	100.0	100.0	100.0	8.7	100.0	100.0	21.7	4.4	82.6	73.9	43.5	87.0
MSSA	44	100.0	100.0	86.4	27.3	100.0	100.0	52.3	9.1	95.5	77.3	75.0	79.5
ST5-t002	4	100.0	100.0	100.0	0	100.0	100.0	0	0	75.0	100.0	100.0	100.0
ST6-t701	4	100.0	100.0	100.0	0	100.0	100.0	100.0	0	100.0	100.0	75.0	75.0
ST22-t309	8	100.0	100.0	25.0	0	100.0	100.0	75.0	0	100.0	87.5	87.5	87.5
ST188-t189	4	100.0	100.0	100.0	0	100.0	100.0	100.0	0	100.0	100.0	50.0	75.0
Others	24	100.0	100.0	100.0	50.0	100.0	100.0	37.5	16.67	95.83	62.5	70.8	75.0
Total	104	100.0	99.0	94.23	13.5	100.0	100.0	27..9	4.8	89.4	76.9	43.3	86.5
*P* values*		–	1.000	0.0047	0.0007	–	–	<0.0001	0.1598	0.1126	1.000	<0.0001	0.9810

*Comparison between MRSA and MSSA isolates

For MSSA, all isolates tested were positive for *icaA*, *icaD*, *clfA*, *clfB*. Only two strains didn’t harbor *ebpS*. The prevalence rate of *fnbpA, fnbpB, cna, bbp, fib, sdrC, sdrD,sdrE* ranged from 9.1% to 86.4%. All isolates of ST59-SCC*mec* IV-t437 MRSA clone didn’t harbour *fnbpB* and *bbp* genes.

Statistically significant differences of *fnbA, fnbB, cna, sdrD* between MRSA and MSSA were found (*P* < 0.05). However, only *fnbpA* was more likely to be presented in MRSA, other significantly different genes were more likely to be present in MSSA.

## Discussion

This study provided important information on the clinical and molecular epidemiology and biofilm formation ability of SA isolated from pediatricians in China. To our knowledge, this is the first study to report the biofilm production of SA clinical strains isolated from Chinese children.

We found that SA infections were more inclined to affect infants. Children under 3 years of age accounted for 74.0% of the total cases with SA infections in the present study. This result was similar to the study reported by Iwamoto et al., which showed that 39.0% of the total 876 pediatric cases were among infants [31]. Furthermore, Suryadevara et al. estimated population-based incidence of invasive SA infection in children <19 years of age (1996 to 2006), and found that the incidence of MSSA and MRSA infections was highest in children 0 to 4 years of age [32]. The reason why infants are more likely to be infected may be due to that infants are frequently colonized by SA, and the carriage of SA was highest in the first three months of life (25.4%) [33], whereas nasal carriage of SA is an important risk factor for SA infection [34]. In addition, infants were more likely to occur community associated infections compared with older children in our study.

Our results revealed that ST59-SCC*mec* IV-t437 was the most prevalent clone both in CA- and HA- MRSA isolates. In this study, the prevalence rate of MRSA ST59 clone (76.7%) was much higher than previously reported (35.8%, MRSA strains were isolated from Chinese children from 2004 to 2012) [11]. What’s more, we need to note that although ST59 was the predominant clone in the MRSA isolates, ST239 clone also accounted for 22.0% in the previous research [11]. However, ST239 was disappeared in our study. ST59 and ST239 were usually community associated and hospital associated clones in China, respectively [35, 36]. The increasing prevalence rate of ST59 and the disappear of ST239 suggested the significant penetration of CA-MRSA clone into hospitals, and even replaced HA-MRSA clone. Indeed, many studies have indicated that CA-MRSA clones are beginning to replace HA-MRSA clones as the predominant cause of hospital infections around the world, such as USA, Greece, Denmark, Uruguay, Korea, Tunisia, and Algeria [37].This maybe due to that CA-MRSA clone carries a shorter SCC*mec* (usually type IV and V) than HA-MRSA clone (usually type I, II and III), which believed to minimized the fitness cost [38]. In addition, *pvl* may be involved because CA-MRSA clones were more likely to carry *pvl*, but *pvl* negative CA-MRSA strains can also cause outbreaks in healthcare settings [39]. Further studies are still needed on this issue.

For MSSA clinical strains, there were diverse genotypes and no dominant clone was identified. The top three MLST types were ST22 (20.5%), ST5 (11.4%) and ST398 (11.4%), which differed from those detected in other regions, such as Europe and Australia [40, 41]. In addition, the most frequent MLST types of MSSA clinical isolates in this study were also different from previous research which showed that ST88, ST25, ST7, ST2155, and ST188 were the top five MLST types for MSSA strains isolated from Chinese children [42]. These results indicate that the molecular characteristics of MSSA may also have regional characteristics, and the common genotypes are also changing with time. Therefore, molecular epidemiological investigations of MSSA strains are also very important, and have great significance to control MSSA clinical infection in a given region.

CA-MRSA clones are usually considered susceptible to most antibiotics other than methicillin and beta-lactams [43]. But in our study, ST59-SCC*mec* IV- t437 clone, the most prevalent clone both in CA-MRSA and HA-MRSA isolates, showed relative high resistant rates to erythromycin, clindamycin, tetracycline, chloramphenicol, and even ciprofloxacin. What’s more, the MDR rate of this clone had reached 83.8%. These results were consistent with a previous research which demonstrated that resistance to non-β-lactams, especially to clindamycin, was high in CA-MRSA isolates from Chinese children, and the MDR rate for ST59 clone was 67.9% [44]. Multi-resistant CA-MRSA clone has also been reported in other countries. For example, CA-MRSA USA300 isolates are becoming more resistant to a variety of antibiotics, including erythromycin, levofloxacin, mupirocin and tetracycline, and have spread to Europe, South America and Australia [45]. This phenomenon should arouse the attention of clinicians when making treatment protocols for patients potentially infected with these bacteria. In addition, MSSA isolates were more susceptible to cefuroxime, clindamycin, and tetracycline than MRSA isolates. But the resistance rate of MSSA to penicillin and erythromycin reached also nearly 100%, which indicated that penicillin and erythromycin may not be suitable for Chinese children with SA infection.

Furthermore, our data demonstrated that the biofilm formation abilities of SA strains are generally high: 83.3% of MRSA and 54.5% of MSSA showed SBF. The generally high biofilm production of SA strains obtained from Chinese children call for greater attention in the treatment of SA infectious diseases, especially indwelling medical device infection. We also found that MRSA strains could produce significantly higher biofilm than MSSA strains. This result was consistent with Kwon et al. describing that the rate of biofilm positivity in MRSA strains was significantly higher than in MSSA strains (37.9% vs. 14.3%, *P* < 0.05) [46]. The morphological studies of Jones et al. also indicated that the MRSA biofilm was thicker than the MSSA biofilm [47]. However, many other studies failed to establish a link between oxacillin resistance and biofilm formation ability [48–50]. Different results of these studies may be due to the following reasons. Firstly, the predominant clone of MRSA has regional characteristics, and MRSA strains can express either low level heterogeneous resistance or high-level, homogeneous resistance to methicillin [51]. These phenomena make the relationship between methicillin resistance and biofilm formation become more complicated. Secondly, the mechanisms of biofilm formation of MRSA and MSSA are different, biofilm formation ability of MRSA and MSSA maybe influenced by the expression level of their respective regulatory mechanism. Researches have shown that MSSA strains form PIA-mediated biofilms whereas MRSA strains form biofilms independent of PIA, but requiring surface proteins and firmly regulated by accessory gene regulator (*agr*) system [51]. Further studies are still needed to explore the relationship between methicillin resistance and biofilm formation ability.

In addition, our results showed that a correlation between the clonal lineage and biofilm formation might be existed. What need to be stressed was that 83.8% of the ST59-SCC*mec* IV-t437 clone, the most prevalent clone of MRSA, showed SBF. The ability of ST59-SCC*mec* IV-t437 clone to form strong biofilm may contribute to its dominance and multi-drug resistance in China. What’s more, all MSSA strains belonging to ST188-t189 showed especially strong biofim formation ability. Although we found that MRSA could produce significantly higher biofilm than MSSA, the extremely high biofilm formation ability of MSSA ST188-t189 isolates indicated that biofilm formation might be more closely related with clonal lineage. The relationship between clonal lineage and biofilm formation has been supported by several other studies. Naicker et al. [50] found that MLST CC5 might be associated with high biofilm formation. Croes et al. [52] also demonstrated that strains associated with MLST CC8 were markedly more often classified as strong biofilm former. Furthermore, Atshan et al. [53] found that isolates belonging to similar *spa*, SCC*mec*, and MLST types had similar abilities to produce biofilms, and isolates of different *spa* types showed high variation in their ability to produce biofilms. These researches, including ours, suggest that clonal lineage might be good predictors of biofilm production.

To understand the molecular mechanism of SA biofilm formation, we detected the frequency of 12 selected genes in biofilm formation. In the present study, all isolates harbored *icaA*, *clfA* and *clfB*, and only one strain didn’t harbor *icaD*. Similar to our study, several other studies also reported a high prevalence rate of these genes [54, 55]. A comparative analysis between MRSA and MSSA isolates regarding the presence of all tested genes showed that *fnbpA* were more inclined to be present in MRSA, whereas *fnbpB*, *cna*, *sdrD* were more likely to be present in MSSA. However, a previous study didn’t find any correction between methicillin resistance and the prevalence of biofilm associated genes [51]. This discrepancy may be due to that specific clonal complexes of SA may contain a unique combination of surface-associated and regulatory genes [56], and the distribution of clonal lineage have regional characteristics. Further researches are still needed to evaluate the expression of these genes in SA.

## Conclusions

In summary, our results indicated strong homology of the MRSA stains isolated from Chinese children, in which multiresistant ST59-SCC*mec* IV-t437 clone with strong biofilm formation ability was determined predominantly. The MSSA strains, in contrast, were very heterogeneity. The generally high MDR rate and biofilm production of SA in this study should arouse the attention of pediatrician in China. In addition, significant differences were found between MSSA and MRSA regarding biofilm formation and several biofilm associated genes (*fnbA, fnbB, cna*, *sdrD*), and an correlation between clonal lineage and biofilm formation might also be existed. Investigation of biofilm production and related molecular mechanisms of SA will ultimately promote the treatment of biofilm mediated infections.

## Additional files

Additional file 1: Prevalence of *Staphylococcus aureus* STs among different infections. (A) Distribution of the STs among strains isolated from patients with skin and soft tissue infections; (B) Distribution of the STs among strains isolated from patients with pneumonia; (C) Distribution of the STs among strains isolated from patients with sterile site infections (bloodstream infections and bone and joint infections). (TIFF 7046 kb)

Additional file 2: Raw OD value of all isolates. (XLSX 14 kb)

## Abbreviations

Bbp: Bone sialoprotein-binding protein
CA: Community associated
ClfA: Clumping factor A
ClfB: Clumping factor B
CLSI: Clinical and Laboratory Standards Institute
Cna: Collagen adhesin
EbpS: Elastin binding protein
EUCAST: European Committee on Antimicrobial Susceptibility Testing
FnBPA: Fibronectin-binding proteins A
FnBPB: Fibronectin-binding proteins B
HA: Hospital associated
Ica: Intercellular adhesion
icaR: Ica regulator
MBF: Moderate biofilm production
MLST: Multilocus sequence typing
MRSA: Methicillin-resistant SA
MSCRAMMs: Microbial surface components recognizing adhesive matrix molecules
MSSA: Methicillin-sensitive SA
OD: Optical density
ODc: Cut-off OD value
SA: *Staphylococcus aureus*
SBF: Strong biofilm production
SCC*mec*: Staphylococcal cassette chromosome *mec*
Sdr C, D, E: Serine-aspartate repeat protein C, D, E
*Spa*: staphylococcal protein A gene
WBF: Weak biofilm production
